# HPV Enhances HNSCC Chemosensitization by Inhibiting SERPINB3 Expression to Disrupt the Fanconi Anemia Pathway

**DOI:** 10.1002/advs.202202437

**Published:** 2022-11-16

**Authors:** Zixian Huang, Yongju Chen, Rui Chen, Bin Zhou, Yongqiang Wang, Lei Hong, Yuepeng Wang, Jianguang Wang, Xiaoding Xu, Zhiquan Huang, Weiliang Chen

**Affiliations:** ^1^ Department of Oral and Maxillofacial Surgery Sun Yat‐sen Memorial Hospital Sun Yat‐sen University Guangzhou Guangdong 510120 P. R. China; ^2^ Guangdong Provincial Key Laboratory of Malignant Tumor Epigenetics and Gene Regulation Sun Yat‐sen Memorial Hospital Sun Yat‐sen University Guangzhou Guangdong 510120 P. R. China; ^3^ Medical Research Center Sun Yat‐sen Memorial Hospital Sun Yat‐sen University Guangzhou Guangdong 510120 P. R. China

**Keywords:** cisplatin resistance, Fanconi anemia pathway, head and neck squamous cell carcinoma, human papillomavirus, nanoparticles, SERPINB3

## Abstract

Head and neck squamous cell carcinoma (HNSCC) is the most common malignant tumor of the head and neck, and the prognosis of patients is poor due to chemotherapeutic resistance. Interestingly, patients with HNSCC induced by human papillomavirus (HPV) infection are more sensitive to chemotherapy and display a better prognosis than HPV‐negative patients. The biological relevance of HPV infection and the mechanism underlying chemosensitivity to cisplatin remain unknown. Herein, SERPINB3 is identified as an important target for regulation of cisplatin sensitivity by HPV‐E6/E7 in HNSCC. Downregulation of SERPINB3 inhibits cisplatin‐induced DNA damage repair and enhances the cytotoxicity of cisplatin. In detail, decreasing SERPINB3 expression reduces the USP1‐mediated deubiquitination of FANCD2–FANCI in the Fanconi anemia pathway, thereby interfering with cisplatin‐induced DNA interstrand crosslinks repair and further contributing to HNSCC cell apoptosis. To translate this finding, pH‐responsive nanoparticles are used to deliver SERPINB3 small interfering RNA in combination with cisplatin, and this treatment successfully reverses cisplatin chemotherapeutic resistance in a patient‐derived xenograft model from HPV‐negative HNSCC. Taken together, these findings suggest that targeting SERPINB3 based on HPV‐positive HNSCC is a potential strategy to overcome cisplatin resistance in HPV‐negative HNSCC and improves the prognosis of this disease.

## Graphical abstract

A pH‐responsive RNAi nanoplatform for targeted regulation of SERPINB3 function and effective inhibition of the Fanconi anemia (FA) pathway to promote cancer therapy.

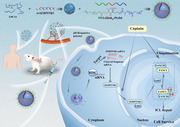

## 1 Introduction

Human papillomavirus (HPV) is a major human carcinogen and causes 4.5% of all cancers worldwide. The most common type is cervical cancer, accounting for almost 100% of cases, and HPV also causes anal cancer (88%), vulvar cancer (25%), vaginal cancer (78%), and penile cancer (50%).^[^ ^1^ ^]^ In particular, head and neck squamous cell carcinoma (HNSCC) caused by HPV infection, especially that found at the base of the tongue and tonsils, is currently increasing in incidence, with positive cases accounting for ≈31% of HNSCC cases.^[^ ^2^ ^]^ Every year, there are more than 600 000 cases of HNSCC, the sixth most common cancer worldwide, but the prognosis of HNSCC is extremely poor due to resistance to radiotherapy and chemotherapy and local recurrence. Hence, its 5‐year survival rate is less than 50%.^[^ ^3^ ^]^ Interestingly, patients with HNSCC caused by HPV infection have a better prognosis than those with HNSCC caused by other factors, with a 5‐year survival rate of 75–80%.^[^ ^4^ ^]^ This favorable prognosis was certified by the Union for International Cancer Control and the American Joint Committee on Cancer, which proposed adding HPV positivity to the 8th edition of the Cancer Staging Manual.^[^ ^5^ ^]^

Studies have suggested that the good prognosis of HPV‐positive HNSCC patients may be due to enhanced sensitivity to treatment‐induced cytotoxicity, including the clinical first‐line chemotherapeutic drug cisplatin.^[^ ^6^ ^]^ After cisplatin enters cells, it reacts with purine residues and forms interstrand crosslinks (ICLs), one of the most cytotoxic types of DNA damage.^[^ ^7^ ^]^ Then, the Fanconi anemia (FA) pathway is activated for ICLs repair in cells. In this pathway, monoubiquitination of the FANCD2–FANCI heterodimer and its deubiquitination mediated by ubiquitin specific peptidase 1 (USP1) are at the core of the FA pathway and the key to repairing ICLs.^[^ ^8^ ^]^ Monoubiquitinated FNACD2‐FANCI localizes to ICLs and recruits downstream FA effectors to promote its repair.^[^ ^9^ ^]^ In addition, the FA pathway needs to coordinate various different processes after the FNACD2‐FANCI reaction, including translesion synthesis (TLS), homologous recombination (HR), and nucleotide excision repair (NER), to ultimately complete ICLs repair.^[^ ^10^ ^]^ If repair fails, subsequent DNA damage cascades will be promoted, followed by genomic instability, chromosomal rearrangements, and DNA double‐strand breaks until cell death occurs.^[^ ^11^ ^]^

The favorable prognosis of HPV‐positive HNSCC may be due to the sensitivity to chemotherapy, which is related to the mechanisms mentioned above. However, due to the presence of more gene mutations and somatic changes, HPV‐negative HNSCC is more resistant to chemotherapeutic drugs and has a poorer prognosis than HPV‐positive HNSCC; these are clinical problems that need to be solved.^[^ ^12^, ^13^ ^]^ It is impossible to improve the prognosis of HPV‐negative HNSCC patients by HPV infection, but we can explore the key mechanisms underlying the sensitivity of HPV‐positive HNSCC to treatment, identify the key regulators, and use the results to develop treatments for HPV‐negative HNSCC.

Herein, we identified SERPINB3, a key factor in the regulation of HPV‐E6 oncogenes, by RNA sequencing (RNA‐seq) based on the cisplatin‐sensitive response of HPV‐positive HNSCC. SERPINB3 is a serine protease inhibitor that is highly expressed in a variety of squamous cell carcinomas and exhibits significant antiapoptotic properties.^[^ ^14^, ^15^ ^]^ We found that SERPINB3 could be downregulated by the HPV oncoprotein E6 and promote cisplatin sensitivity in HPV‐positive HNSCC by inhibiting DNA damage repair. SERPINB3 knockdown inhibited USP1 expression, thereby inhibiting FANCD2–FANCI deubiquitination in the FA pathway. The loss of ICLs repair induced by cisplatin could not be restored, ultimately triggering apoptosis. Therefore, SERPINB3 is a key regulator that is induced by HPV and regulates chemosensitivity in HNSCC.

**Scheme 1:**
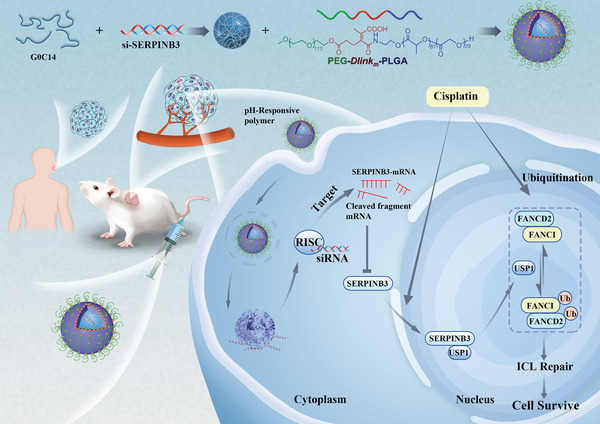
Schematic illustration of a pH‐responsive RNAi nanoplatform for targeted regulation of SERPINB3 function and effective inhibition of the FA pathway to promote cancer therapy. In this study, we found that SERPINB3 can bind USP1; promote monoubiquitination of FANCI and FANCD2, factors in the canonical FA pathway; and accelerate ICL repair triggered by cisplatin. When SERPINB3 was inhibited, the abovementioned regulation of the FA pathway was reduced, thereby increasing tumor sensitivity to cisplatin chemotherapy. We constructed a cisplatin‐resistant PDX model with cells derived from HPV‐negative HNSCC patients. By delivering siSERPINB3 through NPs combined with cisplatin administration, good therapeutic effects were achieved.

Due to the lack of specific inhibitors against SERPINB3, small interfering RNA (siRNA) targeting SERPINB3 is the main method currently used to inhibit SERPINB3 expression. However, naked siRNA is unstable and is easily degraded by enzymes in the blood or phagocytosed by phagocytes in organs, so its ability to reach the tumor site is poor.^[^ ^16^ ^]^ Nanoparticles (NPs), degradable materials for drug delivery, can pass through the blood circulation and become enriched in tumor tissue by the enhanced permeability and retention (EPR) effect.^[^ ^17^ ^]^ Hence, NPs are ideal vehicles for siRNA delivery for targeted gene silencing in vivo. In this research, we developed an endosomal pH‐responsive NP system that targets the unique microenvironment of the tumor to systematically deliver SERPINB3 siRNA to HNSCC tumors in vivo and found that SERPINB3 silencing effectively restored sensitivity to cisplatin in patient‐derived xenografts (PDXs) from HPV‐negative HNSCC patients (**Scheme** **1**). This study suggests that targeting SERPINB3 is a promising strategy to overcome HPV‐negative resistance to chemotherapy in HNSCC.

## 2 Results

### 2.1 HPV‐Positive HNSCC Patients Display a Better Prognosis Than HPV‐Negative HNSCC Patients

Histological analysis of p16 is used to evaluate HPV infection in tumors because p16 expression is highly correlated with HPV infection.^[^ ^18^ ^]^ Combined with HPV‐16/18 E6/E7 mRNA in situ hybridization and E6/E7 protein immunohistochemistry, this method can be used to better distinguish HPV‐positive HNSCC cases.^[^ ^19^, ^20^ ^]^

**Figure 1:**
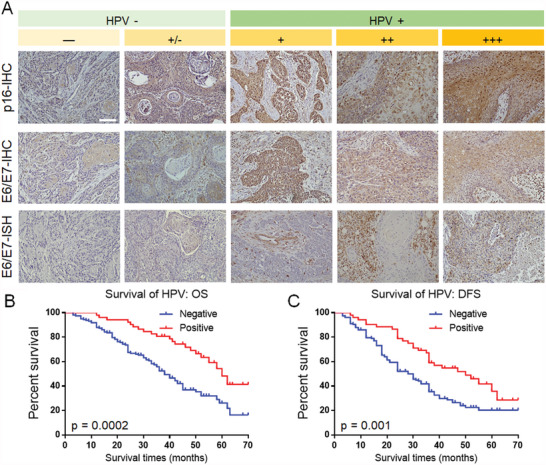
HPV‐positive HNCSS patients display a better prognosis than HPV‐negative HNCSS patients. A) The expression of p16, HPV‐16/18‐E6/E7 in HNSCC tissues. p16, HPV‐16/18‐E6/E7 ISH, and IHC expressions displayed the same trends in HNSCC tissues. Scale bar = 100 µm. B) The overall survival rate of HPV‐positive HNSCC patients is higher than that of HPV‐negative patients. C) HPV‐positive HNSCC patients have higher DFS than HPV‐negative patients.

We analyzed the biopsy tissue samples for p16 and E6/E7 with immunohistochemistry (IHC) and E6/E7 RNA with in situ hybridization (ISH). We observed 21.8% (*N* = 46), 24.2% (*N* = 51), and 23.7% (*N* = 50) positivity for p16, E6/E7 protein, and HPV 16/18 E6/E7 mRNA, respectively, in the samples. The IHC and ISH analyses had an agreement rate of 91.9% (**Figure** **1**A). Therefore, there is a clear correlation between HPV infection and p16 expression (**Table** **1**), and we still use p16 as an indicator of HPV infection in subsequent experiments. We observed differences between the ISH results and the IHC results in 17 patients. We modified the results based on 2/3 positivity. After defining HPV positivity based on the RNA ISH and p16 IHC results, we observed that 24.6% (*N* = 52) of our samples were HPV positive.

**Table 1:** Association of p16 expression with the HPV infection of HNSCC

	p16 Expression			
HPV expression	Low	High	Total	*P‐*value
Negative	157	2	159	<0.01
Positive	8	44	52	
Total	165	46	211	

Based on the clinical characteristics, there was no significant difference in age, gender, differentiation or lymph node metastasis between the HPV‐positive and HPV‐negative patients. However, the HPV‐positive patients showed a greater tendency to exhibit cancer of the oropharynx and the base of the tongue (**Table** **2**). The postoperative overall survival (OS) and disease‐free survival (DFS) of the patients with HPV‐positive HNSCC were longer, suggesting that patients with HPV‐positive HNSCC had a better prognosis than patients with HPV‐negative HNSCC (Figure 1B,C).

**Table 2:** Association of HPV expression with the clinical features of HNSCC patients

	HPV expression		
Clinical characteristic	Negative 159 (75.4%)	Positive 52 (24.6%)	*P*‐value
Age (mean ± SD)	57.3 ± 11.87	56.5 ± 11.95	0.543
≤40	13	6	
40–50	34	6	
50–60	45	23	
>60	67	17	
Sex			0.789
Male	134	43	
Female	25	9	
Location			0.049
Tongue	47	21	
Palate	10	2	
Oropharynx	47	21	
Mouth floor	55	8	
Differentiation			0.159
High	40	13	
Middle	111	30	
Low	8	9	
Lymphatic metastasis			0.614
No	92	28	
Yes	67	24	
Clinical T stage			0.087
1	39	7	
2	49	17	
3	44	15	
4	27	13	

### 2.2 HPV‐E6/E7 Enhances Cisplatin Sensitivity in HNSCC by Inhibiting DNA Damage Repair

**Figure 2:**
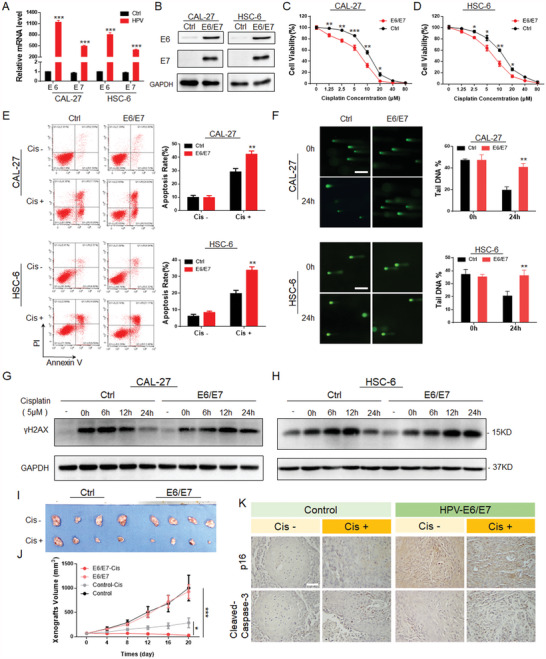
HPV‐E6/E7 increases the sensitivity of HNSCC cells to cisplatin. A) HPV‐E6/E7 proteins were successfully expressed in CAL‐27 and HSC‐6 HNSCC cells, as shown by qPCR detection. B) Immunoblotting detection of HPV‐E6/E7 protein expression in CAL‐27 and HSC‐6 cells. C,D) After transfection, the cell viability in the HPV‐E6/E7 group was lower under treatment with the same concentration of cisplatin. E) Flow cytometry confirmed that the apoptosis rate of the HPV‐E6/E7 group was higher after 5 µm cisplatin treatment for 24 h. F) The alkaline comet assay showed that 24 h after the withdrawal of cisplatin stimulation, comet trailing was still observed in the HPV‐E6/E7 group. Scale bar = 200 µm. G,H) Within 24 h after cisplatin stimulation was withdrawn, *γ*HA2X foci in the HPV‐E6/E7 group were still observed, while the control group had recovered, as shown by immunoblotting detection. I,J) CAL‐27 cells from subcutaneous tumor‐bearing mice with stable HPV‐E6/E7 expression were treated with cisplatin. The HPV‐E6/E7 group displayed the most pronounced decrease in tumor volume. K) The expression of p16 and cleaved caspase‐3 in tumor tissues was histologically analyzed, and the results suggested that there were more apoptotic cells in the HPV‐E6/E7 group. Scale bar = 50 µm. Results are representative of three independent experiments. Data are mean ± SD, *n* = 3, *p < 0.05; **p < 0.01; ***p < 0.001; ns, not significant, paired Student's *t*‐test.

The favorable prognosis of HPV‐positive patients is associated with their enhanced sensitivity to adjuvant chemotherapy. The viral E6 and E7 proteins are the key factors that maintain the malignant phenotype of HPV‐positive cancer cells. Hence, we first expressed E6/E7 in the HNSCC‐negative cell lines CAL‐27 and HSC‐6 by plasmid transfection and constructed HPV‐E6/E7‐overexpressing cell lines. After verification by qPCR and immunoblotting (**Figure** **2**A,B), we found that the E6/E7‐overexpressing cells were ≈2‐fold more sensitive to cisplatin than the control cells (Figure 2C,D). Flow cytometry (FCM) showed that the E6/E7 group had more apoptotic cells after treatment with 5 µm cisplatin (Figure 2E). Subsequently, we constructed a transplanted tumor model with CAL‐27 cells for verification in vivo, and the tumors with E6/E7 overexpression were significantly smaller after cisplatin treatment, and cleaved caspase‐3 as a marker of apoptosis, its expression is significantly increased (Figure 2I–K).^[^ ^21^ ^]^

HPV regulates the cisplatin sensitivity of HNSCC and is related to the repair of DNA damage, which is the cellular response to chemotherapeutic drugs.^[^ ^22^ ^]^ After treatment with cisplatin, *γ*H2AX expression in the E6/E7 group remained high, while that in the control group gradually decreased and returned to the initial level within 24 h after cisplatin was removed (Figure 2G,H). We also observed many *γ*H2AX spots in the E6/E7 group, while the fluorescent spots in the control group had almost disappeared (Figure S1, Supporting Information). In addition, comet tailings were still observed in the E6/E7‐expressing HNSCC cells in the same frame, while tailings in the control group had disappeared, and the fluorescent signal was almost spherical (Figure 2F). The results of the alkaline comet assay confirmed the presence of high levels of damaged and unrepaired DNA in E6/E7 cells, suggesting abnormal DNA damage repair in E6/E7 cells.

Given the above results, we inferred that the HPV oncoprotein E6/E7 promotes the cisplatin sensitivity of HNSCC by disturbing DNA damage repair.

### 2.3 SERPINB3 Is Downregulated in HPV‐Positive HNSCC

**Figure 3:**
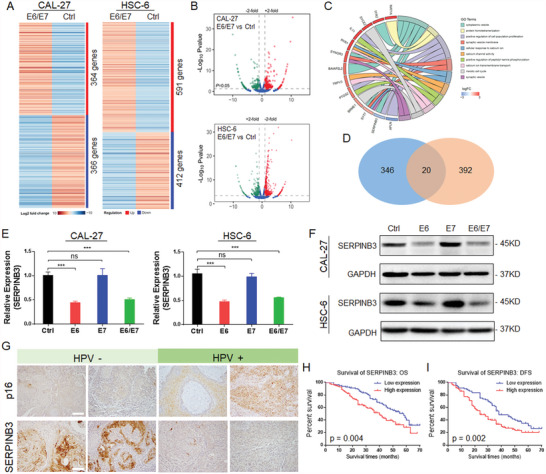
HPV inhibits SERPINB3 expression. A) Heatmap of mRNA sequencing data from HPV‐E6/E7 cells transfected with CAL‐27 and HSC‐6 cells. B) Volcano plot of upregulated (red) and downregulated (green) genes in CAL‐27 and HSC‐6 cells after transfection with HPV‐E6/E7. C) GO analysis of genes downregulated in both cell lines. SERPINB3 is involved in the regulation of multiple signaling pathways. D) Venn diagram showing the intersection of 20 genes downregulated in both cell lines. E) qPCR analysis showed that HPV‐E6 is the key to regulating the inhibition of SERPINB3. F) Immunoblotting showed that the downregulation of SERPINB3 expression was related to HPV‐E6. G) Histological analysis of the relationship between the expression of HPV‐p16 and that of SERPINB3. SERPINB3 expression was downregulated in HNSCC tissues with high p16 expression. Scale bar = 100 µm. H) The overall survival rate of HNSCC patients with low SERPINB3 expression was higher than that of HNSCC patients with high SERPINB3 expression. I) HNSCC patients with low SERPINB3 expression had a higher DFS rate than those with high SERPINB3 expression. Results are representative of three independent experiments. Data are mean ± SD, *n* = 3, *p < 0.05; **p < 0.01; ***p < 0.001; ns, not significant, paired Student's *t*‐test.

The mechanism by which HPV‐E6/E7 regulates the chemosensitivity of HNSCC can be used as a new therapeutic strategy to address cisplatin resistance in HPV‐negative HNSCC. In this regard, we identified SERPINB3 through RNA‐seq and bioinformatics analysis. SERPINB3 was significantly downregulated under E6/E7 induction and found to be associated with cell proliferation and antiapoptotic activity in gene ontology (GO) analysis (**Figure** **3**A–D). Then, we verified the expression of SERPINB3 induced by HPV‐E6/E7 by qPCR and immunoblotting and unexpectedly found that HPV‐E6 led to the downregulation of SERPINB3 expression (Figure 3E,F).

Further histological detection of SERPINB3 was performed in the aforementioned 211 HNSCC patients (Figure 3G, **Table** **3**). We found that among the 52 HPV‐positive cases, SERPINB3 was expressed at low levels in 37 cases (71.2%); among 159 HPV‐negative cases, SERPINB3 was highly expressed in 115 cases (72.3%). Thus, SERPINB3 expression was inversely correlated with HPV infection in HNSCC (Table 3). Patients with lower expression of SERPINB3 displayed longer postoperative OS and DFS (Figure 3H,I). Therefore, we speculated that the HPV oncoprotein promotes the cisplatin sensitivity of HNSCC by inducing the downregulation of SERPINB3.

**Table 3:** Association of SERPINB3 expression with the clinical features of HNSCC patients

	SERPINB3 expression		
Clinical characteristic	Low 81 (38.4%)	High 130 (61.6%)	*P*‐value
Age (mean ± SD)	58.12 ± 11.95	56.47 ± 11.86	0.327
≤40	8	11	
40–50	10	30	
50–60	29	39	
>60	34	50	
Sex			0.456
Male	66	111	
Female	15	19	
Location			0.966
Tongue	25	43	
Palate	4	8	
Oropharynx	31	37	
Mouth floor	21	42	
Differentiation			0.001
High	14	39	
Middle	56	85	
Low	11	6	
Lymphatic metastasis			0.791
No	47	73	
Yes	34	57	
Clinical T stage			0.859
1	20	26	
2	21	45	
3	23	36	
4	17	23	

### 2.4 Downregulation of SERPINB3 Inhibits the Repair of DNA Damage and Enhances Cisplatin Sensitivity in HNSCC

**Figure 4:**
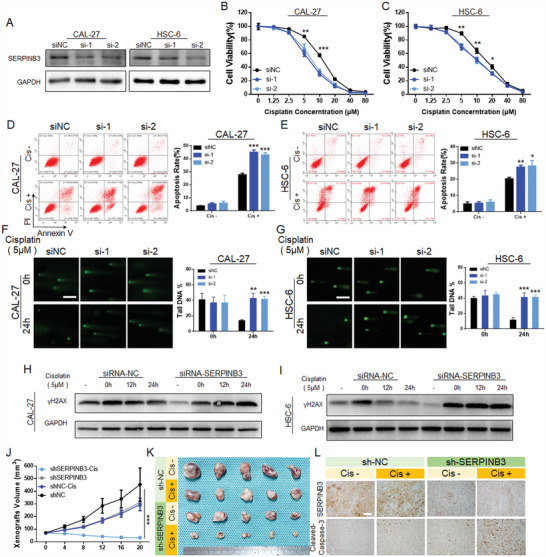
Downregulation of SERPINB3 expression enhances chemosensitivity to cisplatin in HNSCC. A) Immunoblotting confirmed that SERPINB3 protein expression was successfully downregulated. B,C) The cell viability in the SERPINB3‐inhibited group was lower than that in the control group under treatment with the same concentration of cisplatin. D,E) Flow cytometry detection showed that after SERPINB3 was downregulated, the apoptosis rate of cells in the si‐SERPINB3 group was higher under treatment with the same concentration of cisplatin. F,G) The alkaline comet assay showed that 24 h after the withdrawal of cisplatin stimulation, comet trailing was still observed in the si‐SERPINB3 group. Scale bar = 200 µm. H,I) The *γ*HA2X foci in the HPV‐E6/E7 group were still observed, while the control group showed recovery within 24 h after cisplatin stimulation was withdrawn. J,K) A subcutaneous tumor‐bearing model was constructed with the shSERPINB3‐mediated stable SERPINB3 knockdown CAL‐27 cell line. After cisplatin treatment, the tumor growth curve was plotted, and photographs of the collected tumors were obtained (Day 20). In the shSERPINB3 combined with cisplatin group, the inhibitory effect on the tumors was the most obvious. L) Histological detection of SERPINB3 and cleaved caspase‐3 expression in tumor tissues. The results suggested that there were more apoptotic cells in the shSERPINB3‐Cis group than in the control group. Scale bar = 50 µm. Results are representative of three independent experiments. Data are mean ± SD, *n* = 3, *p < 0.05; **p < 0.01; ***p < 0.001; ns, not significant, paired Student's *t*‐test.

For further analysis of the regulatory function of SERPINB3 in HNSCC, SERPINB3 was targeted and inhibited by siRNA (**Figure** **4**A; Figure S2A, Supporting Information). When SERPINB3 was downregulated, HNSCC cells were more sensitive to cisplatin (**Table** **4**). At the same cisplatin concentration, the survival rate of the SERPINB3‐inhibited group was lower (Figure 4B,C), and the proportion of apoptotic cells was higher (Figure 4D,E). Additionally, the clone‐forming capacity of the SERPINB3‐inhibited group was lower (Figure S2B,C, Supporting Information).

**Table 4:** Association of SERPINB3 expression with the HPV infection of HNSCC

	SERPINB3 expression			
HPV expression	Low	High	Total	*P*‐value
Negative	44	115	159	<0.01
Positive	37	15	52	
Total	81	130	211	

Subsequently, the relationship between SERPINB3 and DNA damage repair was verified by the DNA damage marker *γ*H2AX and an alkaline comet assay. Twenty‐four hours after the withdrawal of cisplatin stimulation, there was no significant change in the expression of *γ*H2AX in the SERPINB3‐inhibited group, while the expression of *γ*H2AX in the control group gradually decreased and returned to the prestimulation level (Figure 4H,I). We also observed many *γ*H2AX spots in the SERPINB3‐inhibited group by immunofluorescence (IF) analysis (Figure S2D,E, Supporting Information). Likewise, the results of the alkaline comet assay also supported a delay in the repair of cisplatin‐induced DNA damage in the SERPINB3‐inhibited HNSCC cells (Figure 4F,G). Additionally, we constructed SERPINB3‐overexpressing HNSCC cells by lentiviral infection and verified the enhancement of cisplatin resistance and DNA damage repair (Figure S3, Supporting Information).

A nude tumor‐bearing mouse model was established by the injection of a CAL‐27 SERPINB3 knockdown cell line (shSERPINB3) constructed by shRNA transfection into the right back (Figure S2G, Supporting Information). Cisplatin chemotherapy was administered when the tumor reached 60 mm^3^, and the tumor volume was observed and recorded. The results showed that the tumor volume slightly decreased in the shSERPINB3 group and significantly decreased after cisplatin treatment (Figure 4J,K). Ultimately, low expression of SERPINB3 and increased apoptosis (cleaved caspase‐3) in tumors were confirmed by immunohistochemical analysis (Figure 4L).

Inhibition of SERPINB3 can promote the cisplatin sensitivity of HNSCC. The inhibitory effect of cisplatin on DNA damage repair is similar to that of HPV‐E6/E7. We propose that SERPINB3 is a key factor by which HPV regulates cisplatin sensitivity in HNSCC.

### 2.5 Downregulation of SERPINB3 Inhibits USP1 Regulation of the FA Pathway

**Figure 5:**
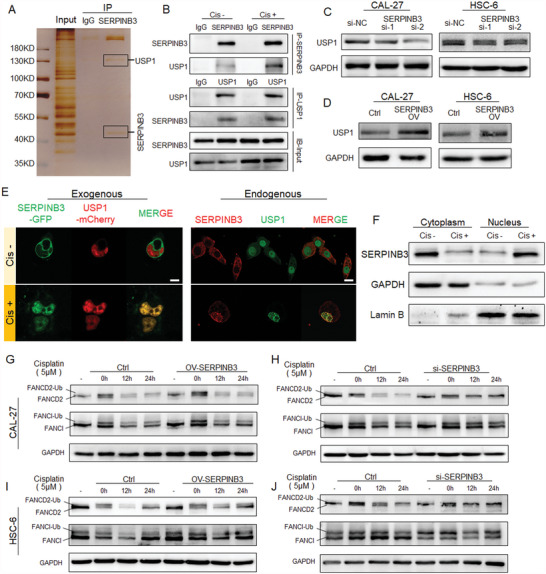
SERPINB3 regulates FANCI and FANCD2 monoubiquitination via USP1. A) SERPINB3 Co‐IP and silver staining showed obvious bands corresponding to the sizes of USP1 and SERPINB3. B) Co‐IP and immunoblotting verified that SERPINB3 and USP1 mutually bind. Their interaction was significantly enhanced after cisplatin treatment. C,D) After SERPINB3 expression was downregulated, USP1 protein expression was downregulated, and vice versa. E) Endogenous and exogenous IF analysis confirmed that after cisplatin stimulation, SERPINB3 entered the nucleus from the cytoplasm and colocalized with USP1. Scale bar = 20 µm. F) Protein nucleocytoplasmic separation was used to verify that SERPINB3 enters the nucleus from the cytoplasm after cisplatin stimulation. G–J) Within the same time frame, after SERPINB3 was overexpressed, the monoubiquitination of FANCI and FANCD2 induced by cisplatin stimulation was quickly activated and then returned to normal levels; by contrast, when SERPINB3 expression was inhibited, the monoubiquitination of FANCI and FANCD2 persisted, suggesting that the FA pathway was abnormal.

The mechanisms of DNA damage repair induced by cisplatin vary and include ICLs, NER, and HR.^[^ ^23^ ^]^ However, the method of SERPINB3 regulation remains unclear. We assessed protein interactions through coimmunoprecipitation (Co‐IP) analysis and protein profiling technology for mechanistic examination. USP1, a key deubiquitination protein, was identified to interact with SERPINB3 (**Figure** **5**A). This protein regulates the deubiquitination of FANCD2 and FANCI and is extremely important for the FA pathway and ICLs repair.^[^ ^24^ ^]^ Inhibiting USP1 leads to the failure of FANCD2 and FANCI deubiquitination, increasing abnormal ICLs repair, causing instability of the genome, and eventually resulting in cellular apoptosis.^[^ ^25^ ^]^

When the expression of SERPINB3 was inhibited or overexpressed, there was no significant difference in the mRNA expression of USP1 (Figure S4, Supporting Information), but the protein expression of USP1 was changed accordingly (Figure 5C,D, Supporting Information). Next, we found that SERPINB3 was normally present in the cytoplasm,^[^ ^26^ ^]^ but it translocated into the nucleus and colocalized with USP1 when HNSCC cells received cisplatin stimulation through exogenous and endogenous methods (Figure 5E,F, Supporting Information). As shown by previous studies, after cells are exposed to UV light, SERPINB3 interacts with the active form of JNK (c‐Jun‐NH2‐terminal kinase) and enters the nucleus from the cytoplasm to exert further antiapoptotic effects on cells.^[^ ^27^ ^]^ We further confirmed by Co‐IP that SERPINB3 and USP1 bind weakly when cells are not stimulated by cisplatin. After cisplatin treatment, SERPINB3 was observed in the nucleus and enhanced its interaction with USP1 (Figure 5B).

Furthermore, we detected FANCD2 and FANCI monoubiquitination (Figure 5G–J). When the stimulus was removed, we found that the ubiquitination of FANCD2–FANCI gradually decreased in the SERPINB3 overexpression group, while the monoubiquitination of FANCD2 and FANCI was maintained in the SERPINB3 inhibition group, and the difference was significant.

Therefore, the downregulation of SERPINB3 inhibits FANCD2–FANCI deubiquitination via USP1, interferes with the FA pathway, and promotes cisplatin sensitivity in HNSCC.

### 2.6 pH‐Responsive NPs Deliver siRNA to Inhibit SERPINB3 Expression

**Figure 6:**
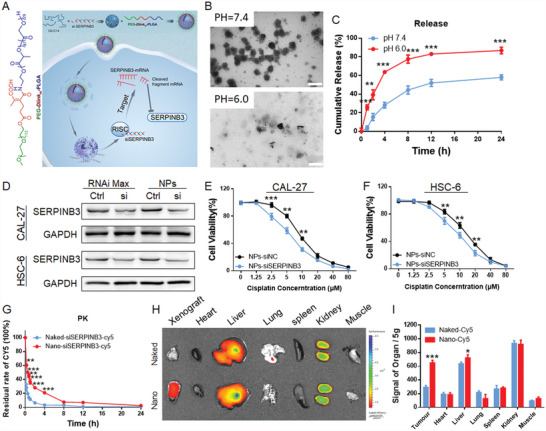
Characteristics and functions of pH‐responsive NPs encapsulated in siSERPINB3. A) Schematic illustration of the role of pH‐responsive NPs in delivering siSERPINB3. B) Morphology of NPs incubated in PBS at 7.4 and 6.0. Scale bar = 200 nm. C) Cumulative siSERPINB3 release from NPs incubated in PBS at different pH values. D) Immunoblotting confirmed the silencing efficiency of NPs compared to that of the previously used RNAi reagent. E,F) NPs inhibited SERPINB3 expression in HNSCC cells, and the cells were significantly more sensitive to cisplatin. G) Blood circulation profiles of naked siSERPINB3 and NP‐encapsulated siSERPINB3. NPs prolonged the siRNA blood circulation. H,I) Biodistribution of naked siRNA and NP‐encapsulated siRNA at 24 h. Tumors and major organs of subcutaneous HNSCC PDX tumor‐bearing mice sacrificed at 24 h post‐injection are shown. The fluorescence images are overlaid, and the siRNA contents in organ homogenates are compared. Results are representative of three independent experiments. Data are mean ± SD, *n* = 3, *p < 0.05; **p < 0.01; ***p < 0.001; ns, not significant, paired Student's *t*‐test.

In the above study, we confirmed that HPV enhances the chemosensitivity of HNSCC to cisplatin via the SERPINB3/USP1/FA pathway. Targeted inhibition of SERPINB3 in HPV‐negative HNSCC may be a feasible strategy to reverse cisplatin resistance in HNSCC. Due to the current lack of molecular inhibitors of SERPINB3, the delivery of siRNA through NPs for SERPINB3 interference is a potential solution. Therefore, we used the pH‐responsive NP delivery system PEG‐D*link_m_*‐PLGA developed by our research group to encapsulate SERPINB3 siRNA for targeted delivery (**Figure** **6**A).

We detected the particle size, potential, morphology, and other characteristics of the synthesized NPs. The results showed that the average size of our encapsulated NPs was 97 nm, and the electric potential was −6 mV (Figure S5A,B, Supporting Information). Transmission electron microscopy of the particle morphology revealed a good nanovesicle structure under neutral conditions (pH 7.4).

After incubation of these siSERPINB3‐loaded NPs in PBS at pH 6.0, the protonated portion of the NPs rapidly dissociated, leading to a change in morphology and the production of large amorphous aggregates with a small particle size (Figure 6B). Within the same time period, the NPs showed obvious release under acidic conditions (pH 6.0), and at 24 h, the amount of siRNA released was close to 85% (Figure 6C). Cell uptake experiments also confirmed that NPs containing encapsulated siSERPINB3 escaped from the lysosome and were released into the cytoplasm within 8 h (Figure S5C, Supporting Information).

We confirmed that the efficiency of NP‐mediated inhibition of SERPINB3 was comparable to that of our above transfection reagents (Figure 6D). After knockdown of SERPINB3 expression by NPs, HNSCC cells were more sensitive to cisplatin (Figure 6E,F). Overall, we confirmed the performance of NPs, the efficiency of target gene knockdown and the effect on the inhibitory function of SERPINB3.

### 2.7 NP‐Delivered siSERPINB3 Enhances Cisplatin Sensitivity in HPV‐Negative HNSCC

With the above promising in vitro results, we finally investigated whether this RNA interference (RNAi) nanoplatform could effectively deliver siRNA into tumor tissues to silence SERPINB3 expression and reverse HPV‐negative PDX chemoresistance.

The pharmacokinetics were first examined by the intravenous injection of NPs loaded with Cy5‐siSERPINB3 into healthy NSG mice (1 nmol of siSERPINB3 per mouse, *n* = 3). As shown in Figure 6G, due to protection by the PEG outer layer, the NPs exhibited a much longer time in the blood circulation than naked siSERPINB3, and more than 10% of the injected NPs remained in the blood at 12 h post‐injection. With this long characteristic circulation time, the NPs could accumulate at high levels in the tumor tissues after intravenous injection into the PDX mice (Figure 6H). Examination of the fluorescence intensity of the collected tumor tissues revealed that the NPs accumulated in the tumors at a level ≈2.5‐fold greater than that of naked siSERPINB3 (Figure 6I).

**Figure 7:**
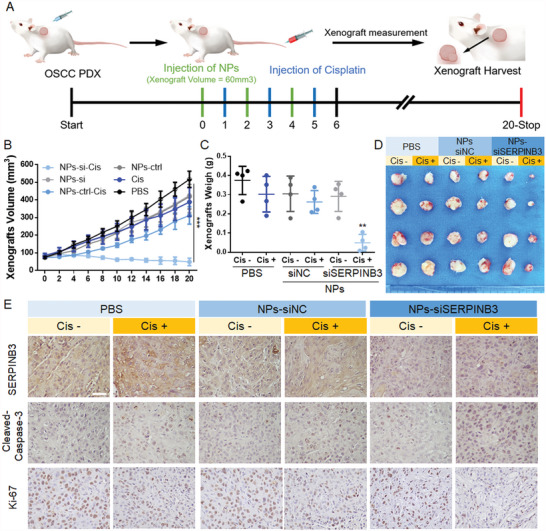
NPs combined with cisplatin in the treatment of an HPV‐negative PDX model. A) Schematic diagram of the treatment of an HNSCC‐negative PDX model. Three cycles of intravenous injection of NPs and cisplatin were applied when the tumor volume was ≈60 mm^3^, and observation continued for 20 days. B) Tumor proliferation in each group. The inhibition of tumor growth in the combination (NPs‐siSERPINB3‐Cis) group was significantly increased compared to that in the other groups. C) Comparison of tumor weight on the last day (Day 20). D) Photograph of collected xenografts on Day 20. Among the groups, the NPs‐siSERPINB3‐Cis group displayed the smallest xenografts. E) Histological detection of SERPINB3, cleaved caspase‐3, and Ki‐67 expression in the tumor tissues. The results suggested that there were more apoptotic cells in the NPs‐siSERPINB3‐Cis group whose proliferative abilities were inhibited. Scale bar = 50 µm. Results are representative of three independent experiments. Data are mean ± SD, *n* = 5, *p < 0.05; **p < 0.01; ***p < 0.001; ns, not significant, paired Student's *t*‐test.

With these promising in vivo results, the NPs were intravenously injected into HPV‐negative PDX model animals (Figure S6, Supporting Information) to evaluate their ability to reverse chemoresistance (**Figure** **7**A). As shown in Figure 7B–D, after three consecutive injections of NPs‐siSERPINB3 and cisplatin, tumor growth was significantly inhibited, and tumor size even decreased within 20 days. By contrast, the mice treated with control NPs with/without cisplatin showed a more than fivefold increase in tumor size from ≈70 to ≈588 mm^3^. The improved anticancer effect of siSERPINB3 combined with cisplatin compared to that of control NPs was further demonstrated by histological analysis (Figure 7E), which revealed that SERPINB3 was effectively inhibited and that cleaved caspase‐3 expression was increased and ki‐67 was decreased in the tumor tissues.

Moreover, the administration of NPs did not influence the weight of the PDX model mice (Figure S6B,C, Supporting Information), indicating low in vivo toxicity of NPs. To further evaluate the potential in vivo side effects, we performed routine blood analysis and found that aminotransferase, aspartate aminotransferase, total protein, alkaline phosphatase, blood urine nitrogen, and creatinine were also in the normal range (Figure S7A, Supporting Information). Histological analysis showed that there were no observable histological changes in the heart, liver, spleen, lung, or kidney tissues (Figure S7B, Supporting Information). All these results indicated the low in vivo toxicity of the NPs developed in this work.

## 3 Discussion

This study is based on the sensitivity of HPV‐positive HNSCC to cisplatin therapy. We aimed to identify the key factors that regulate chemosensitivity via HPV oncogenes and clarify the signaling pathways involved. Based on its ability to address cisplatin resistance, the identified factor was then applied in HPV‐negative HNSCC to promote cisplatin sensitivity. Herein, we found that SERPINB3 is downregulated by HPV‐E6 and reverses the cisplatin resistance of HPV‐negative HNSCC by interfering with DNA damage repair of the FA pathway by inhibiting USP1, thereby providing a theoretical basis for clinical targeted therapy.

SERPINB3, a member of the serine protease inhibitor (serpin) superfamily subtype B, is related to the occurrence and development of multiple types of squamous cell carcinomas, including uterine cervix carcinoma, esophagus carcinoma, HNSCC.^[^ ^28^ ^]^ Indeed, the overexpression of SERPINB3 can significantly enhance the antiapoptotic activity of cancer cells by inhibiting cell damage.^[^ ^29^, ^30^ ^]^ Under the induction of DNA crosslinking agents, SERPINB3 can have a cytoprotective effect by inhibiting lysosomal damage and further enhancing the chemoresistance of cancer cells.^[^ ^30^ ^]^ Our results showed that SERPINB3 participates in the repair of DNA damage induced by cisplatin. After the expression of SERPINB3 was knocked down in vitro, repair was significantly reduced, and resistance to cisplatin‐induced apoptosis was decreased, thus promoting cisplatin sensitivity in HNSCC. As a key protein in HPV‐mediated cancer transformation, E6 can inactivate a number of cellular proteins that contain PDZ domains, such as the tumor suppressor p53,^[^ ^31^ ^]^ human discs large tumor suppressor,^[^ ^32^ ^]^ and members of the MAGI family of proteins,^[^ ^33^ ^]^ which are dependent on the ubiquitin ligase UBE3A/E6AP‐mediated proteasomal degradation pathway.^[^ ^34^ ^]^ Whether SERPINB3 inhibition after HPV‐E6 induction depends on the ubiquitin‐mediated mechanism above remains unknown. More work needs to be done in the future.

The sensitivity of tumors to cisplatin mainly depends on the ability of cisplatin to recognize and repair DNA damage.^[^ ^35^, ^36^ ^]^ Based on the discovery and confirmation that SERPINB3 regulates the cisplatin sensitivity of HNSCC through DNA damage repair, we identified USP1, which interacts with SERPINB3, by Co‐IP and mass spectrometry. USP1 is a member of the deubiquitinating enzyme family that can bind USP1‐associated factor to form a protein complex and participate in the regulation of DNA damage responses, mainly the FA pathway and TLS repair.^[^ ^37^, ^38^ ^]^ Our study found that SERPINB3 in HNSCC cells could undergo cytonuclear translocation and colocalize with USP1 after treatment with cisplatin. This result is similar to that of Katagiri Chika,^[^ ^27^ ^]^ who found that SERPINB3 could exert an antiapoptotic effect in cells by binding phosphorylated JNK1 and entering the nucleus with JNK1 after UV irradiation. Therefore, we propose that SERPINB3 is involved in the DNA damage response by regulating the expression of USP1.

The FA pathway plays a crucial role in ICLs repair induced by cisplatin.^[^ ^39^ ^]^ Once ICLs occurs, ATR/CHK1 signaling is activated, forming the FA complex, which binds the E2‐binding enzyme UBE2T and monoubiquitinates FANCD2 and FANCI at K561 and K523, respectively.^[^ ^40^ ^]^ The monoubiquitinated FANCD2–FANCI heterodimer localizes to the ICLs and recruits nucleases, polymerases, and downstream proteins to cooperate in ICLs repair.^[^ ^41^ ^]^ During this process, USP1‐mediated FANCD2–FANCI deubiquitination is essential for progression of the FA pathway and ICLs repair.^[^ ^42^ ^]^ The knockdown or loss of USP1 will lead to the failure of FANCD2–FANCI deubiquitination, DNA repair disorders and genomic instability, which eventually lead to long‐term arrest of cells in S phase and cell death.^[^ ^43^ ^]^ We found that SERPINB3 expression was inhibited, and USP1 expression was suppressed, thus leading to abnormal deubiquitination of FANCD2–FANCI. However, after the overexpression of SERPINB3, the expression level of USP1 was increased, which could accelerate the deubiquitination of FANCD2–FANCI during ICLs repair. Therefore, we deduced that the knockdown of SERPINB3 inhibited the deubiquitination of FANCD2–FANCI by USP1 and resulted in the failure of ICLs repair, thereby enhancing the cytotoxicity of cisplatin.

The poor prognosis of HNSCC patients after chemotherapy is related to resistance to platinum drugs, the effects of which include the enhancement of DNA damage repair, antioxidative stress, and NF‐*κ*B signaling pathway activation.^[^ ^44^, ^45^, ^46^ ^]^ In this study, SERPINB3 displayed the potential to reverse cisplatin resistance in HNSCC. Given the current lack of targeted drugs for SERPINB3, nanobiotechnology has shed new light on the application of new therapeutic targets for cancer.^[^ ^47^ ^]^ We can precisely deliver siRNA to the tumor and efficiently knock down the target gene by the EPR effect with NPs. At present, a variety of NPs have been approved by the FDA for tumor treatment or are under preclinical or clinical investigation.^[^ ^48^, ^49^, ^50^ ^]^ Herein, we developed a pH‐responsive NP to carry SERPINB3 siRNA combined with cisplatin for HPV‐negative PDX model treatment, which resulted in the effective downregulation of SERPINB3 in a PDX tumor model and exhibited a promising synergetic antitumor effect.

## 4 Conclusion

In summary, we have identified a key regulator, SERPINB3, from HPV‐positive HNSCC that is downregulated by HPV and moderates the chemoresistance of HNSCC by affecting USP1 and the FA pathway. As a proof‐of‐principle study, we translated this mechanism to the treatment of an HPV‐negative HNSCC PDX model by NP‐delivered siSERPINB3, which effectively enhanced the efficacy of cisplatin treatment. Taken together, the results of this study may provide a new target for HPV‐positive HNSCC treatment. Furthermore, the combination of RNAi technology and a nanodrug delivery system suggests a new strategy for the comprehensive treatment of HNSCC. As a next step, further research will expand the scope of application of this study's conclusions, such as in the treatment of other HPV‐negative tumors and multidrug‐resistant HNSCC.

## 5 Experimental Section

### Antibodies and Reagents

Antibodies specific for DYKDDDDK (FLAG tag, #14793) and *γ*H2AX (#80312) and mouse IgG‐HRP (#7076) were purchased from Cell Signaling Technology, USA. Antibodies specific for GAPDH (sc‐47724) and HPV16‐E7 (sc‐6981) and mouse antirabbit IgG‐HRP (sc‐2357) were purchased from Santa Cruz Biotechnology, USA. Antibodies specific for HPV16/18‐E6 (ab70) were purchased from Abcam, USA. Antibodies specific for SERPINB3 (26558‐1‐AP), USP1 (14346‐1‐AP), FANCD2 (28619‐1‐AP), FANCI (67304‐1‐Ig), and KI67 (27309‐1‐AP) were purchased from Proteintech, China.

Chemicals were purchased from Sigma. All of the culture media (DMEM, DMEM‐F12) and fetal bovine serum (FBS) were purchased from Biological Industries, China.

### Plasmids

Plasmids harboring the human HPV16‐E6/E7, SERPINB3, and USP1 sequences were generated by IGE Biotechnology (Guangzhou, China) using the pCDH‐CMV‐MCS‐EF1‐Puro vector. SERPINB3 shRNA with the following sequence was purchased from Vigene Biosciences: GAGCTGACTTCGGAACTAATTCAAGAGATTAGTTCCGAAGTCAGCTCTTTTTT. pMD2.G (#12259, Addgene) and psPAX2 (#12260, Addgene) were used as packaging vectors. The FLAG, SFB, EGFP, and mCherry tags were added before the N‐terminus or C‐terminus according to a design prepared using HIFI methods. All of the constructs were confirmed by both DNA sequencing and diagnostic digestion.

### HNSCC Sample Collection and Patient Follow‐Up

For this study, patients who presented at the Department of Oral and Maxillofacial Surgery at Sun Yat‐sen Memorial Hospital between 2015 and 2018 for the treatment of HNSCC were recruited. The inclusion criteria were a pathological diagnosis of SCC and willingness to participate in the subsequent follow‐up. Patients were excluded as study subjects if they had been diagnosed with multiple cancers or other severe diseases. HNSCC patient characteristics, including age, sex, tumor location, differentiation, lymphatic metastasis, and clinical stage, were collected and categorized following the 8th Edition TNM Staging and 2022 NCCN guidelines. All patients had a referral at least every season. In addition, tumor samples and adjacent noncancerous (ANC) samples were collected. The ANC tissues were at least 2 cm from the tumor lesion, representing the resection border, and were pathologically confirmed as noncancerous tissues.

### Cell Culture and Transfection

The human HNSCC cell lines CAL‐27, HSC‐6, and HEK293t were obtained from the American Type Culture Collection. All cell lines were routinely cultured in DMEM with 10% FBS in a 37 °C humidified incubator containing 5% CO_2_. All the cell lines were validated by short tandem repeat profiling analysis and were free of mycoplasma contamination.

For transient transfection using siRNAs, siRNAs targeting SERPINB3 and USP1 were synthesized by IGE Biotechnology (Guangzhou, China). Transfection was performed with Lipofectamine RNAiMAX reagent (Invitrogen, USA) according to the manufacturer's protocol. The siRNA sequences were as follows:

siRNA sequences for SERPINB3 and EGFR

**Table:** 

Gene		Forward	Reverse
siNC		UUCUCCGAACGUGUCACGUdTdT	ACGUGACACGUUCGGAGAAdTdT
SERPINB3	s1	GGUUCUUCACUUUGAUCAAdTdT	UUGAUCAAAGUGAAGAACCdTdT
	s2	GAAAGUCGAAAGAAGAUUAdTdT	UAAUCUUCUUUCGACUUUCdTdT
USP1	s1	CUGCCAUCAUUAUACUGAAdTdT	UUCAGUAUAAUGAUGGCAGdTdT
	s2	GAUGCAUAGUGGCAUUACAdTdT	UGUAAUGCCACUAUGCAUCdTdT

For stable expression, lentiviral plasmids harboring the desired gene or shRNA were first transfected into 293T cells together with the packaging plasmids pSPAX2 and pMD2.G at a ratio of 5:3:2. HEK293 cells were placed in a 10 cm plate and cultured as previously described. After reaching 70–80% confluence, the cells were transfected with 6 µg of psPAX2, 3 µg of pMD2.G, and 10 µg of transfer vector using Lipofectamine 3000 reagent. Forty‐eight hours after transfection, the supernatants of each group were collected and used to infect HNSCC cells for another 48 h. Puromycin‐tolerant cells were selected. Subsequent western blotting and PCR were applied to confirm correct expression in the stable cell lines.

### RNA Extraction and RT‐PCR

Total RNA was extracted using TRIzol reagent (TaKaRa, Japan) according to the manufacturer's instructions and then reverse transcribed into cDNA using PrimeScript RT Master Mix (TaKaRa, Japan) on an ABI 9700 Real‐Time PCR system (ABI, USA). The newly synthesized cDNA was then used as a template for detection of the desired gene.

Specifically, 1 µL of cDNA was mixed with TB Green Premix Ex Taq II (TaKaRa, Japan) in a 20 µL reaction volume. All of the reactions were run in triplicate using the primers described above. The reaction conditions were as follows: 94 °C for 2 min and 40 cycles of 94 °C for 20 s, 58 °C for 20 s, and 72 °C for 20 s. The relative expression of mRNA was detected using the Roche LightCycler 480 II Real‐time PCR machine (Roche, USA). The primer sequences were as follows:

Primer sequences for PCR

**Table:** 

Gene	Forward	Reverse
GAPDH	GAGTCAACGGATTTGGTCGT	GACAAGCTTCCCGTTCTCAG
HPV‐E6	GCACAGAGCTGCAAACAACTATACA	TCCCGAAAAGCAAAGTCATATACC
HPV‐E7	GCATGGAGATACACCTACATTG	TGGTTTCTGAGAACAGATGG
SERPINB3	CGCGGTCTCGTGCTATCTG	ATCCGAATCCTACTACAGCGG
USP1	GCTGCTAGTGGTTTGGAGTTT	GCATCACAACCGCAAATAATCC

### Western Blotting, Co‐IP, and Mass Spectrometry

For protein extraction, the cells were washed twice with cool PBS, harvested by scraping and then lysed in lysis buffer (#9803e, CST). Following centrifugation, the supernatant was collected, and the protein concentration was determined using a BCA Kit (Pierce, Thermo).

For western blotting, cell lysates were electrophoretically separated on an SDS‐PAGE gel using a standard protocol. The proteins were then transferred to polyvinylidene fluoride membranes (IPVH00010, Millipore). The membranes were blocked with 3% nonfat milk in Tris‐buffered saline containing 0.1% Tween‐20 (TBST) for 1 h at room temperature. The blots were then incubated with the antibodies mentioned above at 4 °C overnight, washed in TBST and then probed with secondary antibody. Western blot analysis was performed using the statistical gray values from the blots.

For immunoprecipitation, the supernatants were first incubated with S‐protein agarose beads (#69704, Millipore, for SFB) overnight at 4 °C, and the precipitates were washed three times with lysis buffer. For detection of endogenous interactions, the clarified supernatants were incubated with the antibodies mentioned above for 2 h and then with magnetic beads (#88802, Thermo Fisher) overnight. After being washed three times with lysis buffer, the samples were collected and analyzed by western blotting.

Proteins immunoprecipitated with SFB‐SERPINB3 were harvested by Co‐IP and detected by silver staining using a silver staining kit (#G7210, Solarbio). Additionally, the proteins were digested into peptides and then analyzed by mass spectrometry.

### Cisplatin Cytotoxicity Assay

The appropriate cells were plated in 96‐well plates (10 000 cells per well) and incubated overnight. Then, the cells were treated with cisplatin (#1134357, Sigma) at the indicated concentrations (0, 2.5, 5, 10, 20, 40, 80, and 160 µmol L^−1^), and the CCK‐8 (#ab228554, Abcam) assay was applied to examine the cytotoxicity of cisplatin after 48 h of treatment. The supernatant was removed, and 100 µL of a mixture of 10% CCK‐8 reagent in DMEM was added. The plates were incubated for an additional 2 h, and the absorbance at 490 nm was measured using a microplate reader (Multiskan MK3, China).

The relative cell survival (%) was determined by the following formula: (OD × *χ* µmol/L/OD 0 µmol/L) × 100, where *χ* represents the cisplatin concentration.

The percentage of cell mortality (%) was then calculated as 100 – relative cell survival.

### Cell Proliferation Assay

At 24 h after transfection, the cells were collected, and 2000 cells per well were plated in 96‐well plates. The numbers of cells at 24, 48, and 72 h were determined using the MTS Assay Kit (#G3580, Promega, USA). The medium was removed from each well, and 100 µL of 10% MTS in DMEM was added. The plates were incubated for an additional 2 h, and the absorbance at 490 nm was measured using a microplate reader (Multiskan MK3, China). The data are presented as the original OD values.

### Colony Formation Assay

Two hundred transfected cells per well were placed in 6‐well plates and cultured for 14 days, with the medium replaced every 5 days. Colonies were fixed with methanol and stained with 0.1% crystal violet in PBS for 15 min. Colony formation was determined by counting the number of stained colonies.

### FCM Analysis

For the flow cytometric quantification of cell death, the cells were treated as indicated. Then, the cells were collected, washed twice with PBS and stained using annexin V‐FITC/PI (#556547, BD) according to the manufacturer's instructions. Stained cells were analyzed using a Becton Dickinson FACScan flow cytometer (FACScan, BD), and data were processed using FlowJo software.

### Alkaline Comet Assay

A total of 3 × 10^5^ transfected cells per well were seeded in a 6‐well plate and incubated overnight. Cells in the indicated wells were treated with cisplatin (5 µm mL^−1^) for 24 h. Then, the culture medium was removed and replaced, at which point the time was set as 0 h; the cells were then collected at 0 h or incubated for 24 h and collected. Alkaline comet assays were carried out according to the instructions of a single‐cell gel electrophoresis kit (#4250‐050‐ES, R&D Systems). DNA images were obtained with a fluorescence microscope, and comet score analysis of the images was performed to obtain the DNA tail‐to‐tail ratio.

### IF Analysis and Confocal Imaging

For detection of *γ*H2AX, SERPINB3 and USP1 in HNSCC cells, 1 × 10^4^ cells were plated on glass‐bottomed dishes (#D35‐20‐0‐TOP, Cellvis). The cells were then treated with cisplatin (5 µm mL^−1^) for 24 h. Then, the medium was removed and replaced with culture medium, and the cells were incubated for 24 h before collection.

After fixation with 4% paraformaldehyde and permeabilization with 0.3% Triton X‐100, the cells were blocked in 0.5% BSA–PBS for 1 h. Primary antibodies (*γ*H2AX, SERPINB3, USP1, 1:100) were used for incubation with the cells at 4 °C overnight. The next day, after washing with PBST, the cells were stained with secondary antibodies (1:500) and DAPI (1:1000). Fluorescence images were taken on a confocal laser microscope (Zeiss LSM 800).

For time‐lapse imaging (NP uptake) of HNSCC cells, GFP‐endo14‐labeled CAL‐27 cells at ≈30–40% confluency were seeded on glass‐bottomed dishes for 16 h. After the medium was replaced with 2 mL of fresh medium, NPs loaded with Cy5‐labeled siSERPINB3 were added at a siRNA concentration of 50 nm, and the cells were allowed to incubate for 8 h during confocal capture.

The captured images were analyzed with the Zen Blue microscope imaging software (Zeiss) or ImageJ program.

### Preparation of NPs

The amphiphilic polymer PEG‐Dlinkm‐PLGA was synthesized according to the previous study and then dissolved in *N*,*N*′‐dimethylformamide (DMF) to form a homogenous solution with a concentration of 20 mg mL^−1^. Then, a mixture of 1 nmol of siSERPINB3 (0.1 nmol µL^−1^ in ddH_2_O) and 50 µL of G0‐C14 (5 mg mL^−1^ in DMF) was prepared and mixed with 200 µL of PEG–Dlinkm–PLGA solution. Under vigorous stirring (1000 rpm), the mixture was added dropwise to 5 mL of deionized water. The NP dispersion was transferred to an ultrafiltration device (100 kDa MWCO, Millipore) and centrifuged to remove the organic solvent and free compounds. After two washes with deionized water, the obtained NPs were dispersed in 1 mL of deionized water. The size and zeta potential were determined by dynamic light scattering (Malvern Zetasizer). The morphology of the saporin‐loaded NPs was visualized on a Tecnai G2 Spirit BioTwin transmission electron microscope. Before observation, the samples were stained with 1% uranyl acetate and dried under air.

For determination of the siRNA encapsulation efficiency (EE%), Cy5‐labeled siSERPINB3 was encapsulated into the NPs according to the method described above. Subsequently, 10 µL of the NP solution was mixed with 20‐fold dimethylsulfoxide (DMSO). The standard was prepared by mixing 10 µL of naked Cy5‐siSERPINB3 (1 nmol mL^−1^) with 20‐fold DMSO. The fluorescence intensity was measured using a multimode microplate reader (Tecan SPARK 10M), and the vector EE was calculated as follows: EE% = (FINP/FIStandard) × 100. NPs with an EE% greater than 80% were adopted for further experiments.

### In Vitro siRNA Release

NPs loaded with Cy5‐si SERPINB3 were prepared as described above. Subsequently, the NPs were dispersed in 1 mL of PBS (pH 7.4) and then transferred to a Float‐a‐lyzer G2 dialysis device (100 kDa MWCO, Spectrum) that was immersed in PBS (pH 6.0) at room temperature. At a predetermined interval, 10 µL of the NP solution was withdrawn and mixed with 20‐fold DMSO. The fluorescence intensity of Cy5 was determined using a microplate reader as described above.

### In Vitro Gene Silencing

HNSCC cells were seeded in 12‐well plates (50 000 cells per well) and incubated in 1.5 mL of culture medium (pH 7.4) with 10% FBS for 24 h. Thereafter, the medium was replaced with fresh medium, and NPs loaded with siSERPINB3 were added at a siRNA concentration of 50 nm, which was the same concentration of RNAiMAX used. After 24 h of incubation, the cells were washed with PBS (pH 7.4) and allowed to incubate in fresh medium (pH 7.4) for another 48 h. After removal of the medium and three washes with PBS buffer (pH 7.4), the cells were collected for qPCR and immunoblot analysis of SERPINB3 expression.

### Pharmacokinetics Study

Healthy female NSG mice were randomly divided into two groups (*n* = 3) and given an intravenous injection of either naked Cy5‐siSERPINB3 or NPs encapsulating Cy5‐siSERPINB3 at 1 nmol of siRNA/200 µL per mouse. At predetermined time intervals, orbital vein blood (20 µL) was withdrawn using a tube containing heparin, and the wound was pressed for several seconds to stop the bleeding. The fluorescence intensity of Cy5‐labeled siRNA in the blood was determined with a microplate reader.

### Biodistribution

Tumor‐bearing NSG mice were randomly divided into two groups (*n* = 3) and given an intravenous injection of either naked Cy5‐siSERPINB3 or NPs encapsulating Cy5‐siSERPINB3 at a 1 nmol siRNA dose per mouse. Twenty‐four hours after the injection, the mice were imaged using the IVIS system (Cri, Inc.). The organs and tumors were then harvested and imaged. For quantification of the accumulation of NPs in the tumors and organs, the tissues were homogenized, and the fluorescence intensity of Cy5 in each organ was examined with a microplate reader. The Cy5 intensity is presented as (total intensity)/organ weight.

### In Vivo Tumor Xenograft

For determination of the effects of HPV‐E6/E7 and SERPINB3 on HNSCC tumors in vivo, CAL‐27 cells infected with HPV and stable shSERPINB3‐expressing cell lines were used to establish tumor xenografts. A total of 2.0 × 10^6^ transfected CAL‐27 cells were implanted into the right upper backs of BALB/c nude mice. The tumors were measured every 4 days to determine the tumor volume (mm^3^), which was equal to length × width^2^ × 0.5. When the tumor volume reached ≈60 mm^3^, 3 mg kg^−1^ cisplatin was intraperitoneally injected every 4 days into mice in the treatment group. Tumor volume was observed, and growth curves were constructed. The mice were sacrificed 10 days after three injections for a total of 21 days of observation. The tumors were harvested and then paraffin embedded for IHC detection.

### PDX Model and NP Application

For generation of a PDX model of HPV‐negative HNSCC, tumor tissue was obtained from HPV‐negative HNSCC patients, cut into small pieces and subcutaneously transplanted into the right upper back of NSG (NOD/SCID/IL2R*γ* null) mice (female, 5 weeks, 16–18 g). When the tumor volume reached ≈60 mm^3^, NPs combined with cisplatin were prepared for treatment. The NPs with 10 nm siSERPINB3 were administered by tail vein injection every 2 days for a total of three times. Cisplatin (3 mg kg^−1^) was administered as described above to nude mice for combined drug application. The mice were sacrificed 10 days after the treatments for a total of 21 days of observation. Then, the tumors were harvested for IHC detection. The organs and peripheral blood were harvested for toxicity detection.

### RNA ISH

Experiments were conducted with formalin‐fixed, paraffin‐embedded biopsy tissues and analyzed as 5‐µm‐thick sections (HNSCC patients, *N* = 211). Slides were deparaffinized by baking in a 60 °C oven, followed by xylene immersion prior to tissue staining. RNA ISH was conducted to detect HPV. Specifically, E6/E7 mRNA was detected from HPV types 16 and 18 by a digoxigenin‐labeled probe (E6‐CTCACGTCGCAGTAACTGTTGCTTGCAGTACA; E7‐TGGACCATCTATTTCATCCTCCTCCTCTGAGCT). After deparaffinization, the tissue sections were treated according to the digoxigenin‐labeled in situ hybridization kit instructions (ISH‐7001, ZSGB‐BIO, China).

### Immunohistochemistry

Immunohistochemical staining was performed according to standard protocols. After deparaffinization, antigen retrieval was conducted using Tris‐EDTA buffer (#C1038, Solarbio) in a microwave oven for 15 min. Briefly, the tissue sections were blocked sequentially with 3% H_2_O_2_ and normal serum and then incubated with primary antibodies at 4 °C overnight. The tissues were incubated with a biotinylated secondary antibody and conjugated with a streptavidin‐HRP complex (ready‐to‐use SP kit; Zhongshan Co., China). Finally, the sections were visualized with 3‐3′‐diaminobenzidine, counterstained with hematoxylin and mounted. The samples were rinsed with PBS between each step.

### Evaluation of IHC and ISH Staining

Following IHC staining, the tissues were evaluated by two pathologists who assessed the number of positive cells and staining intensity. Positive staining was assessed using a semiquantitative scoring system. The percentage of positive cells was scored as follows: 0 (no staining), 1 (<1/3 staining), 2 (1/3 to 2/3 staining), and 3 (>2/3 staining). Staining intensity was scored as follows: 0 (negative), 1 (weak positive), 2 (medium positive), and 3 (strong positive). The final evaluation of protein expression was based on the sum of the scores for percentage positivity and intensity. A score of 0–2 was defined as negative, while a score >2 was defined as positive. For RNA ISH, the manufacturer's scoring guidelines were followed such that positive staining was defined as having at least 1 positive signal per cell in tumor regions.

### Statistical Analysis

All statistical analyses were conducted using SPSS 19.0 statistical software. Spearman correlation analyses and an unpaired *t*‐test were used to examine the correlation between HPV‐P16, DNA‐HPV ISH, RNA‐HPV‐E6/E7 ISH, and SERPINB3 expression in HNSCC patients. Kruskal–Wallis analysis was used to examine the relationships between clinicopathological characteristics and protein expression. Survival curves were plotted using the Kaplan–Meier method and compared with the results of the log‐rank test. Student's *t*‐test was used to compare differences in gene expression levels determined by PCR, cell apoptosis, tumor xenograft volumes, and cell functions (proliferation, colony formation, etc.) between the different groups. Unless otherwise noted, quantitative data are expressed as the mean and standard deviation (S.D.). Statistical significance was determined with a paired Student's *t*‐test (^∗^ *p* < 0.05; ^∗∗^ *p* < 0.01; ^∗∗∗^ *p* < 0.001, compared with the control).

### Ethical Statement

This research was conducted in accordance with international guidelines and the ethical standards outlined in the Declaration of Helsinki. This study was approved by the Sun Yat‐sen Memorial Hospital Institutional Review Board. All animal experiments were conducted following the Ministry of Health national guidelines for the housing and care of laboratory animals and performed in accordance with institutional regulations after review and approval by the Institutional Animal Care and Use Committee at Sun Yat‐sen University (2022000537).

## Conflict of Interest

The authors declare no conflict of interest.

## Supporting information

Supporting Information

Supporting Information

Supporting Information

## Data Availability

The data that support the findings of this study are available from the corresponding author upon reasonable request.
