# Supplementary material for: HPV Enhances HNSCC Chemosensitization by Inhibiting SERPINB3 Expression to Disrupt the Fanconi Anemia Pathway
Source: Adv Sci (Weinh). 2022 Nov 16;10(1):2202437. doi: 10.1002/advs.202202437 (PMC9811475; doi:10.1002/advs.202202437)
Supplement: Supplementary file 1 — Supporting Information [file ADVS-10-2202437-s001.pdf]

ADVANCED SCIENCE
Open Access

Supporting Information

for *Adv. Sci.*, DOI 10.1002/advs.202202437

HPV Enhances HNSCC Chemosensitization by Inhibiting SERPINB3 Expression to Disrupt the Fanconi Anemia Pathway

Zixian Huang, Yongju Chen, Rui Chen, Bin Zhou, Yongqiang Wang, Lei Hong, Yuepeng Wang, Jianguang Wang, Xiaoding Xu, Zhiquan Huang* and Weiliang Chen**

Figure S1

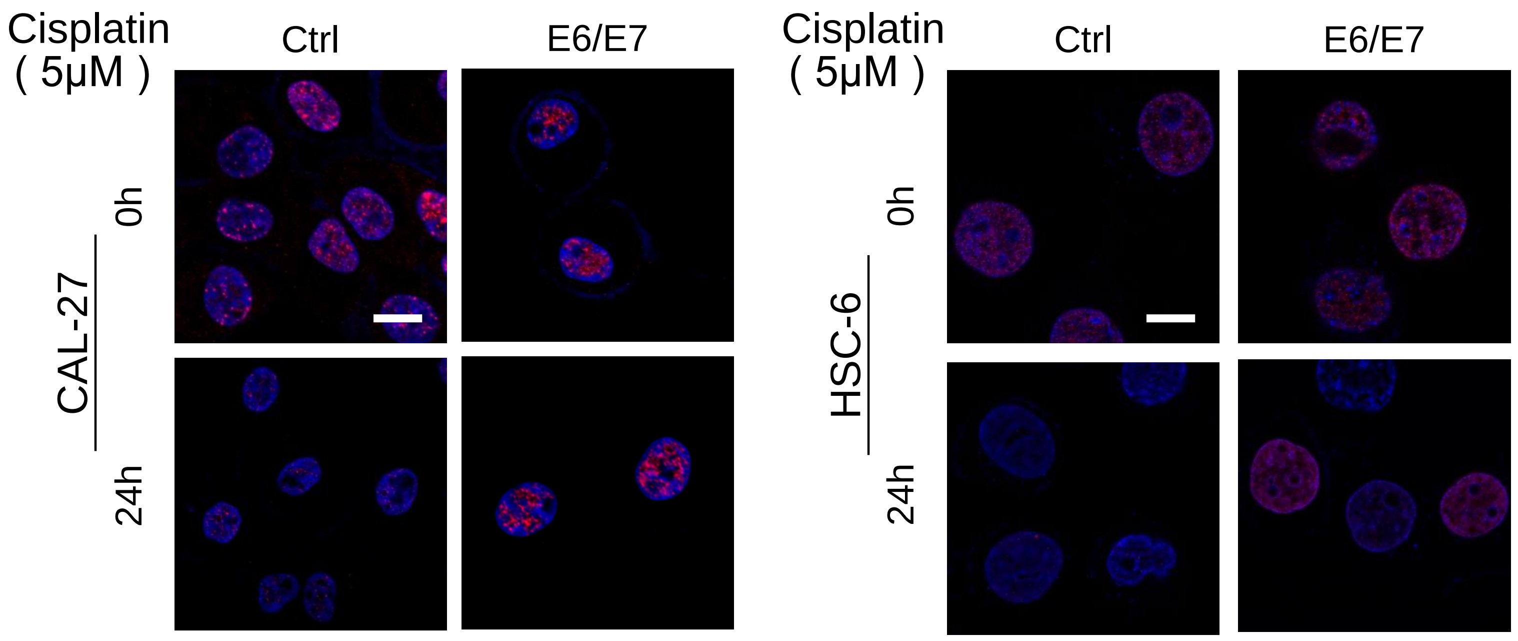

Figure S1

After 24 h of cisplatin stimulation and the withdrawal of cisplatin, fluorescent γHA2X foci were still observed in the E6/E7 group, while the fluorescent spots in the control group had almost disappeared. Scale bar = 5um

Figure S2

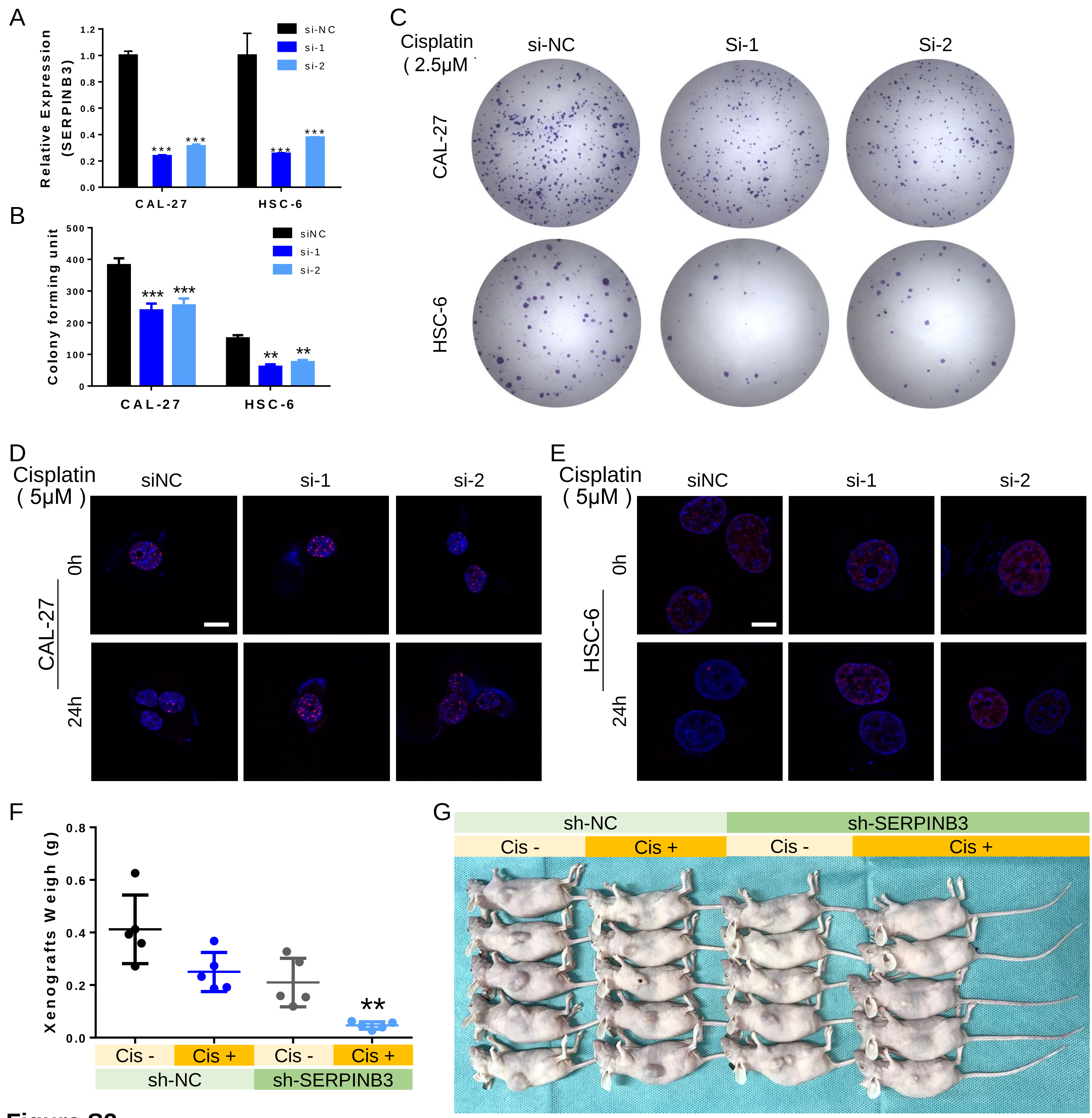

Figure S2

A. qPCR detection confirmed that the mRNA expression of SERPINB3 in CAL-27 and HSC-6 cells was downregulated.

B & C. After the expression of SERPINB3 was inhibited, the rate of cell clone formation was significantly reduced under stimulation with a low concentration of cisplatin (2.5 μM).

D & E. After 24 h of cisplatin stimulation and the withdrawal of cisplatin, fluorescent γHA2X foci were still observed in the siSERPINB3 group, while the fluorescent spots in the control group had almost disappeared. Scale bar = 5 um

F. Comparison of tumor weights in the mice. The sh-SERPINB3-Cis group had the smallest tumor weight.

G. Mice from each group.

Results are representative of 3 independent experiments. Data are mean ± SD, n=3(A&B), n=5(F) $*p < 0.05$; $**p < 0.01$; $***p < 0.001$; ns, not significant, paired Student's t-test.

Figure S3

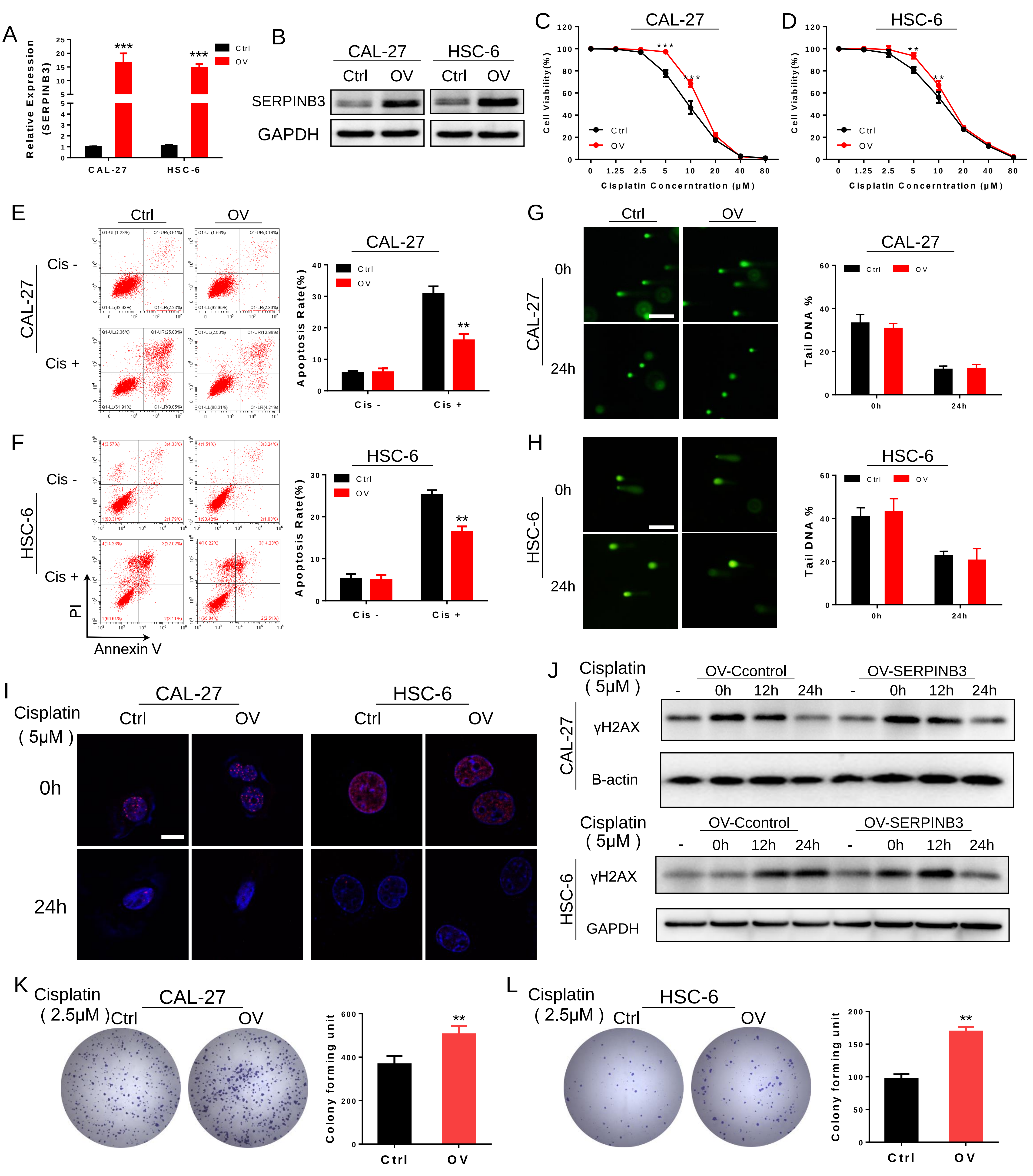
A
Relative Expression (SERPINB3)
Ctrl
OV
***
CAL-27
HSC-6
B
CAL-27
HSC-6
Ctrl
OV
SERPINB3
GAPDH
C
CAL-27
Cell Viability(%)
Cisplatin Concerntration (μM)
D
HSC-6
E
Cis -
Cis +
Apoptosis Rate(%)
F
PI
Annexin V
G
0h
24h
Tail DNA %
H
I
Cisplatin (5μM)
J
OV-Ccontrol
OV-SERPINB3
12h
γH2AX
B-actin
K
Cisplatin (2.5μM)
Colony forming unit
L

Figure S3

A. qPCR detection confirmed that SERPINB3 was overexpressed in HNSCC cells.

B. Western blot detection confirmed that SERPINB3 was overexpressed in HNSCC cells.

C & D. The cell viability in the SERPINB3-overexpressing group was higher than that in the control group under treatment with the same concentration of cisplatin.

E & F. Flow cytometry detection showed that the apoptosis rate of cells in the SERPINB3-overexpressing group was lower than that in the control group under treatment with the same concentration of cisplatin.

G & H. The alkaline comet assay showed that 24 h after the withdrawal of cisplatin stimulation, comet trailing in the SERPINB3-overexpressing group had disappeared. Scale bar = 200 μm

I. After 24 h of cisplatin stimulation and the withdrawal of cisplatin treatment, the fluorescent γHA2X foci had almost disappeared in the SERPINB3-overexpressing group. Scale bar = 5 μm

J. γHA2X foci were restored in the SERPINB3-overexpressing group within 24 h after cisplatin stimulation was withdrawn.

K & L. Under stimulation with a low concentration of cisplatin (2.5 μM), the rate of SERPINB3-ov cell colony formation was significantly higher than that of the control cells.

Results are representative of 3 independent experiments. Data are mean ± SD, $n = 3$, *$p < 0.05$; **$p < 0.01$; ***$p < 0.001$; ns, not significant, paired Student’s t-test.

Figure S4

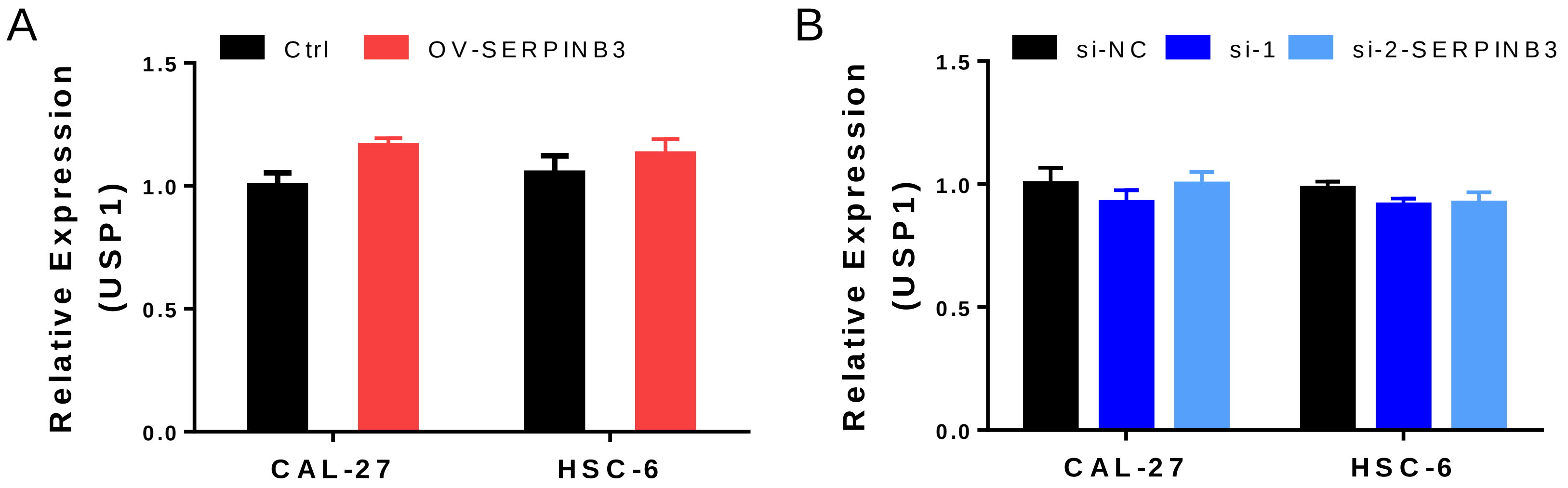

Figure S4

A. The mRNA expression of USP1 did not change significantly after overexpression of SERPINB3.

B. After the expression of SERPINB3 was inhibited, the mRNA expression of USP1 did not change significantly.

Figure S5

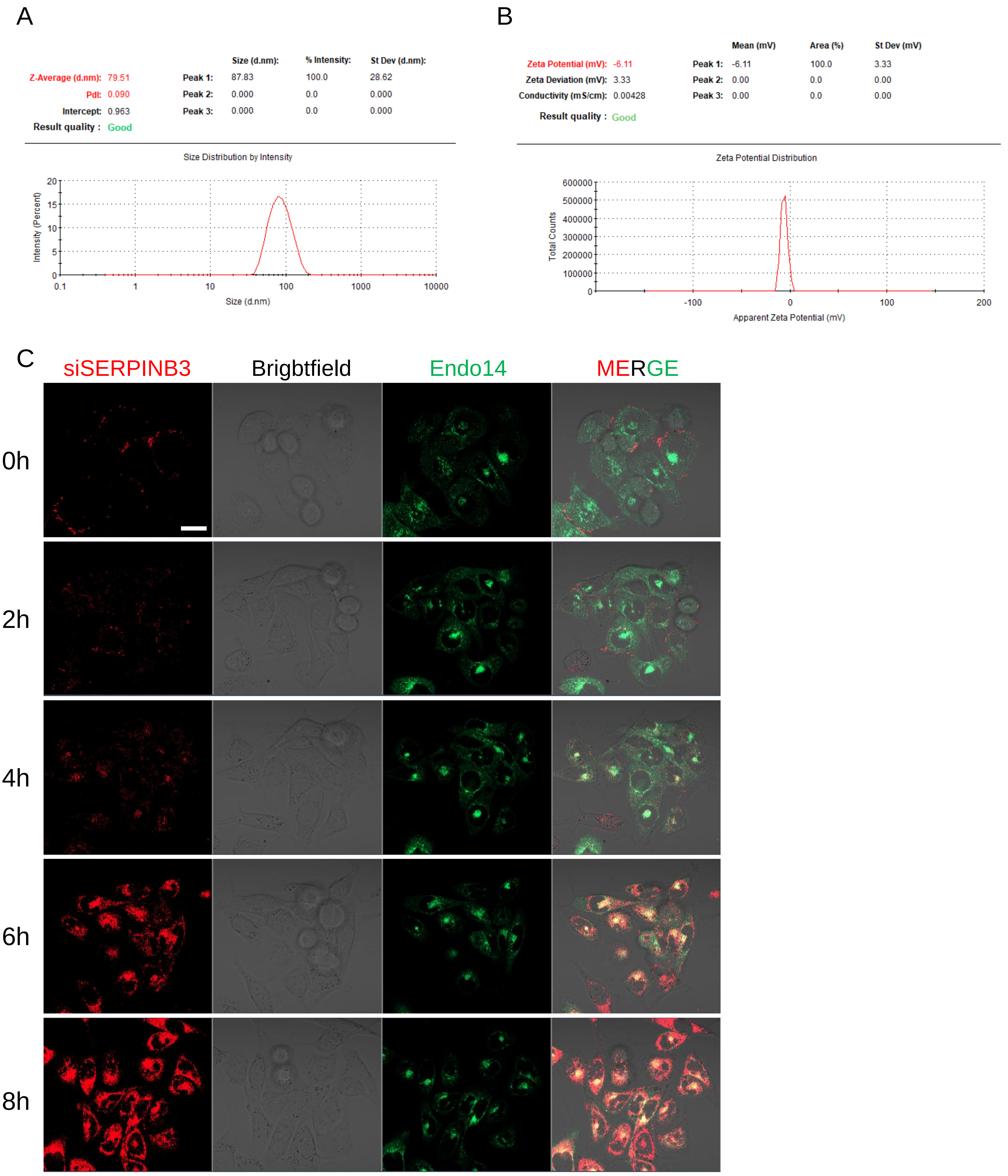

Figure S5

A. The size of NPs containing encapsulated siSERPINB3.

B. The zeta potential of NPs containing encapsulated siSERPINB3.

C. When NPs loaded with siSERPINB3-Cy5 were added to stably transfected HSC 6 Endo14-GFP cells, the NPs entered the cells after 4 h, were released from the endosomes in 6 h, and filled the entire cytoplasm after 8 h. Scale bar = 10 um

Figure S6

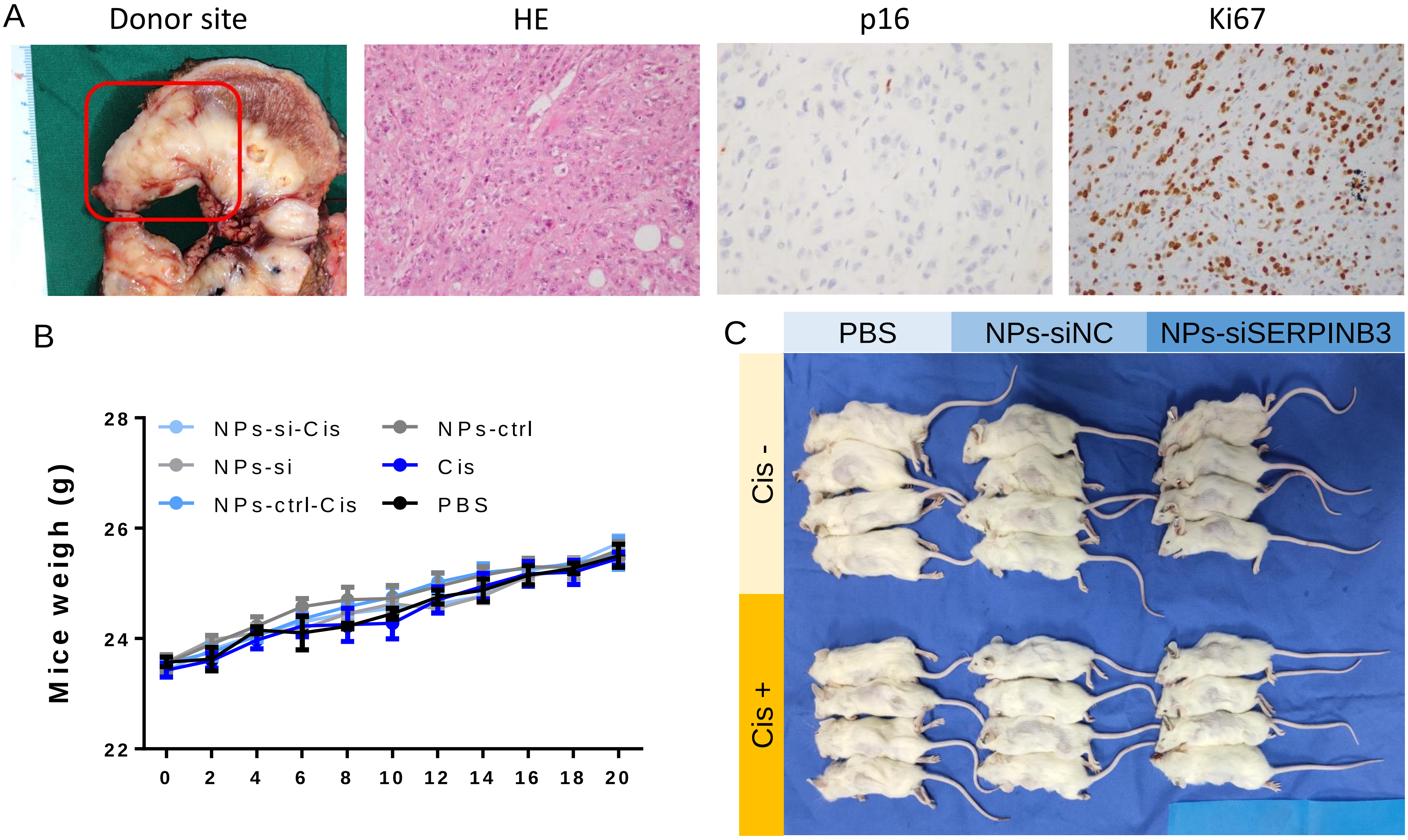

Figure S6

A. Tumor pictures and main results of histological analysis of the HPV-negative HNSCC tissue donor.

B. Changes in the weight of the mice in each group during the treatment; the results suggested that the NPs did not affect the mouse weight.

C. Pictures of the mice in each group.

Results are representative of 3 independent experiments. Data are mean ± SD, n = 5, *p < 0.05; **p < 0.01; ***p < 0.001; not significant, paired Student's t-test.

Figure S7

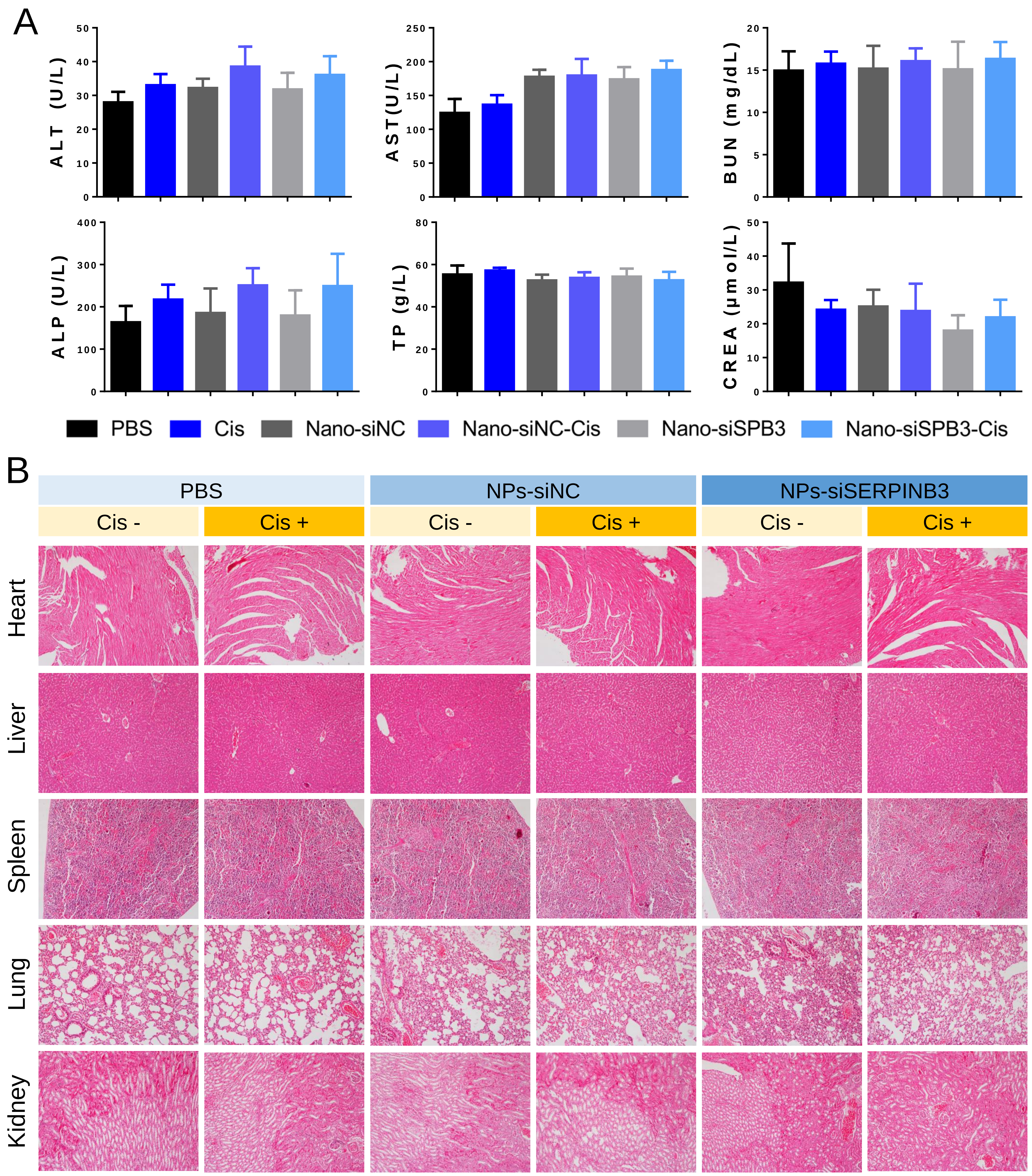

Figure S7

A. Serum levels of alanine aminotransferase (ALT), aspartate aminotransferase (AST), blood urine nitrogen (BUN), alkaline phosphatase (ALP), total protein (TP), and creatinine (CREA) after treatments.

B. Histological staining of sections of major organs from PDX tumor-bearing mice in different treatment groups.

Results are representative of 3 independent experiments. Data are mean ± SD, n = 5, *p < 0.05; **p < 0.01; ***p < 0.001; not significant, paired Student's t-test.
